# Pain, Physical Function, Radiographic Features, and Quality of Life in Knee Osteoarthritis Agricultural Workers Living in Rural Population

**DOI:** 10.1155/2019/7684762

**Published:** 2019-09-29

**Authors:** Gordana Nikolic, Biserka Nedeljkovic, Goran Trajkovic, Dragisa Rasic, Zlatica Mirkovic, Slavica Pajovic, Rade Grbic, Sandra Sipetic, Isidora Vujcic

**Affiliations:** ^1^Internal Clinic Laplje Selo, Clinical Hospital Center Pristina-Gracanica, Prishtina, Serbia; ^2^Pulomonology Clinic, Clinical Hospital Center Pristina-Gracanica, Prishtina, Serbia; ^3^Institute of Medical Statistics and Informatics, Faculty of Medicine, University of Belgrade, Belgrade, Serbia; ^4^Clinic for Orthopaedic, Clinical Hospital Center Pristina-Gracanica, Prishtina, Serbia; ^5^Institute of Epidemiology, Faculty of Medicine, University of Belgrade, Belgrade, Serbia

## Abstract

**Background:**

The aim of this study was to analyse the relationship between the clinical manifestations, disease severity based on radiography images, functional activity level, and quality of life in patients with knee osteoarthritis in a rural population living in Serbian enclaves in Kosovo, as well as to determine the correlation between the WOMAC and the EQ-5D questionnaire in this population.

**Method:**

The cross-sectional study was conducted at the Internal Medicine Clinic, Clinical Hospital Center Pristina-Gracanica, located in Laplje Selo from February to December 2013. One hundred patients with confirmed (American College of Rheumatology criteria) knee osteoarthritis completed the EQ-5D and Western Ontario and McMaster Universities Osteoarthritis Index (WOMAC) questionnaires, rated pain on a visual analogue scale (VAS), and underwent knee radiographic examinations.

**Result:**

Most patients were obese with moderate radiographic changes according to the Kellgeren–Lawrence scale and suffered from very severe pain according to the VAS scale. The duration of disease significantly correlated with the WOMAC scores, VAS score, and all of the scores on the EQ-5D, except for mobility. The age of participants showed a similar correlation with the same variables. The patients with higher Kellgren–Lawrence scores (3-4) were significantly older, with a significantly higher body mass index (BMI) and longer duration of disease than patients with lower scores (1-2). Significantly higher VAS, pain/discomfort EQ-5D, and WOMAC pain and function scores were also recorded among patients with more significant radiological changes. The correlations between WOMAC and EQ-5D were satisfactory.

**Conclusion:**

The severity of clinical manifestations and radiographic area changes may affect functional ability and the quality of life in knee OA patients living in rural areas, which requires adequate treatment and physical therapy.

## 1. Introduction

Knee osteoarthritis (OA) is a chronic, degenerative disease that may occur at any period of life after adolescence, but the prevalence of this disease increases with age. Knee OA is becoming a great public health problem around the world due to increasing life expectancy, and it is considered the leading cause of disability in the general population in people older than 65 [1]. It manifests as joint pain, stiffness, a limited range of motion, crepitations, joint effusions, deformities, bony enlargements, and various grades of inflammation. Pain, as the basic disease symptom, is directly related to the radiographic changes and other symptoms and signs and is a reliable indicator of future disability [2–4].

There is an increasingly greater need to resolve the problem of disease management in knee OA patients, as interventions that delay or stop the disease progression do not exist. The presence of clinical manifestations causes functional deficits and loss of independence when performing everyday activities, depression and the social isolation of the patients, thereby increasing the risk of morbidity and mortality [5–9]. The instruments used to evaluate the quality of life are widely applied to measure pain and physical functioning, as well as mental, health, and social functioning [10, 11]. The EQ-5D is a widely used generic questionnaire for evaluating quality of life and has been shown to be a reliable and valid instrument in patients with knee OA [12], while WOMAC is a specific questionnaire that is frequently given preference over generic questionnaires due to its greater sensitivity in detecting minimal important differences in OA.

The Internal Medicine Clinic, as a facility of the Clinical Hospital Center Pristina-Gracanica, is situated in Laplje Selo; patients from all Serbian enclaves in the Kosovo territory south of the river Ibar (Gračanica, Štrpce, Novo Brdo, Ranilug, Pasjane, Parteš, and Klokot) are treated in this institution. According to the 2011 census results, 25,532 Serbs lived in this area [13].

The purpose of this study was to analyse the relationship between the clinical manifestations, disease severity based on radiography images, functional activity levels, and quality of life in patients with knee OA living in the rural population in Serbian enclaves, as well as to determine the correlation between the WOMAC and EQ-5D questionnaire in this population.

## 2. Materials and Methods

### 2.1. Participants

The cross-sectional study was conducted in 2013 at the Internal Medicine Clinic Laplje Selo. One hundred consecutive patients with OA older than 40 years were included in the study; no one declined participation. The Ethics Committee of the Medical Faculty of the Pristina University which was temporarily settled in Kosovska Mitrovica approved the study. The diagnosis of knee OA was established according to the American College of Rheumatology (ACR) classification criteria [14]. Sample size calculation was based on a regression model with a prespecified number of predictors and coefficients of determination. The minimum required sample size needed to detect significance for the multiple regression model with four predictors, coefficient of determination of 0.2, alpha of 0.05, and power of 0.8, was 53.

Exclusion criteria were conditions leading to lower limb immobility or lower limb abnormality, degenerative processes on other bone-joint structures of the lower limbs and spinal column (excluding the knees), use of nonsteroidal anti-inflammatory drugs immediately prior the examination (up to 7 days prior the conducted research), patients requiring hospital treatment, and patients with any systemic connective tissue disease.

The patients gave written consent prior to study enrolment after they were informed of the purpose and objectives of the research.

### 2.2. Measurement

Radiography of both knees in the anteroposterior and lateral projections was performed for each patient. The radiography images were interpreted and scored for overall radiographic severity using Kellgren–Lawrence grades (0–4) by one radiologist, who was blinded to patients' details. To facilitate data analysis, Kellgren–Lawrence stage 1 or 2 were grouped as early and stage 3-4 as late radiological OA.

For each participant, height and weight were measured and recorded by a rheumatologist; body mass index (BMI) was calculated as weight in kilograms divided by the square of the height in metres. All patients completed the EQ-5D and WOMAC questionnaire (Likert version). The intensity of pain in patients with OA was assessed by using a visual analogue scale (VAS), consisting of a 10 cm-long horizontal line marked with “no pain” on one end, and “worst pain imaginable” on the other end. The patients marked the place that corresponds best to their pain intensity on the given line. The numerical values on the VAS were obtained as the distance in centimeter from “no pain” to the point marked on the line by each patient.

### 2.3. Statistical Analysis

Descriptive statistics were used to describe demographic and clinical characteristics. The relationships of certain characteristics such as age, duration of disease, and BMI with the WOMAC and EQ-5D subscales were determined using the Spearman correlation coefficient (data were not normally distributed). The nonparametric Mann–Whitney *U*-test and parametric Student's *t*-test were used to compare the variables between early (1 or 2) and late (3 or 4) Kellgren–Lawrence radiological stages. Spearman correlation coefficient was performed to determine the relationships between the EQ-5D and WOMAC scores. *p* values < 0.05 were considered statistically significant. The statistical analysis was conducted using the program SPSS (version 17).

## 3. Results

### 3.1. Sample Characteristics

All patients were agricultural workers. The average age of the knee OA patients was ±8.4 years, and 75% of them were female. In more than half of patients, the disease lasted for more than five years. The average value of BMI was 30.0 ± 3.0, which indicates that most of the patients in our study were obese. The majority of patients had moderate degenerative changes in both knees (Table 1).

### 3.2. WOMAC, EQ-5D, and VAS Scores and Their Correlation with Patient Characteristics

Table 2 shows the arithmetic means and standard deviations for certain WOMAC, EQ-5D, and VAS scores in patients with knee OA. A mean VAS score of 7.5 indicated that most of the patients suffer from severe pain. Also, the EQ-5D pain/discomfort mean score was higher than the scores in the four other dimensions.

Patient characteristics (age, BMI, and duration of disease) were checked for possible significant correlations with the WOMAC, EQ-5D, and VAS scores. The age of the subjects was significantly positively correlated with the VAS, WOMAC pain and function, and EQ-5D self-care, pain/discomfort, and anxiety/depression scores. The duration of disease significantly correlated with all scores, except for EQ-5D mobility score. In contrast, the BMI of patients significantly positively correlated only with EQ-5D mobility score (Table 3).

### 3.3. Radiography Features and Other Patient Characteristics

Patients with higher Kellgren–Lawrence scores (3 or 4) were significantly older (*p* < 0.001), with a significantly higher BMI (*p*=0.004) and longer duration of disease (*p* < 0.001) in comparison with patients with a lower Kellgren–Lawrence score (1 or 2). In addition, significantly higher VAS scores (*p* < 0.002), pain/discomfort EQ-5D subscores (*p* < 0.001), and WOMAC pain (*p*=0.041) and function subscores (*p*=0.005) were recorded in the late stage group (Table 4).

### 3.4. Correlation between Instruments

The WOMAC pain and function scores best correlated with the pain/discomfort dimension in the EQ-5D scale. In contrast, WOMAC stiffness score showed no significant correlation only with pain/discomfort dimension in the EQ-5D scale. The correlations between the instruments were satisfactory (Table 5).

## 4. Discussion

The majority of patients in our study had moderate degenerative changes in both knees that were most probably the result of the constant, excessive, and bilateral physical load on the knee joints. Agricultural workers are at increased risk of developing knee osteoarthritis because of the repetitive forceful work that can aggravate and accelerate the development of disease [15]. Certain work activities such as kneeling, squatting, lifting, and climbing especially increase the risk of knee OA [16]. Some studies indicated that both squatting/kneeling and high BMI were independent risk factors for knee OA, but that their combination was particularly harmful and significantly increased the risk of developing disease [16–18]. Systematic review and meta-analysis of prospective studies showed that the risk of knee OA increases by 35% with a 5 kg/m^2^ increase in BMI and that obesity was an independent predictor of knee OA risk regardless of the study country, sample size, gender, duration of follow-up, presence of adjusted knee injury, and quality of the study [19]. Obesity was observed in most of our patients. Therefore, overuse and constant knee load during performing agricultural activities associated with obesity, advanced age, and female gender were significantly associated with an increased risk of radiographic knee OA in our patients. All patients in our study live and work in rural areas and have participated in agricultural activities since their early childhood. Multiple studies have shown a consistent association between years of farming and knee OA [15].

Pain is a key symptom among patients with knee OA that influences the decision to seek medical care and is an important precursor of disability [20]. All three scales used in our study indicated that patients suffered from severe pain. Pain in knee OA patients in our study was significantly correlated with age, duration of disease, and radiographic features according to the Kellgren–Lawrence grading scale. OA is the commonest knee pathology in older people, and radiographs appropriately identify moderate and severe OA [21]. A systematic summary of the literature in which 20 studies were included indicates that knee pain is an imprecise marker of radiographic knee OA, even in older age groups, and that imprecision depends on the extent of the radiographic views of the joint obtained [21]. Although they were moderate, the morphological, progressive, and degenerative changes of the knees in elderly, obese subjects in our study performing heavy farming activities were accompanied by severe pain and significantly influenced everyday activities and quality of life. Some studies suggested that associations between radiographic OA and quality of life may be mediated by pain [21, 22]. In our patients, there is an unjustified opinion that the degenerative process on the joints is part of the normal process of aging, and that patients spend a long time on their own analgesics and go to the physician late.

According to results of our study, radiological severity was correlated with functional disability but not with mobility in knee OA patients. The discordance between radiographic OA and the occurrence of clinical symptoms is well documented [23, 24]. Study conducted in Japan indicated that the EQ-5D utility scores were not significantly associated with the KL grade of the knee after adjustment for age, BMI, and grip strength [25].

Using the WOMAC scale, our study showed a positive correlation between pain severity and disability, which is in accordance with results from other studies [26]. The high level of functional reduction among patients in our study is most probably the result of reporting late to a physician and self-medicating. The patients in our study spared their joints by avoiding physical activity due to discomfort and fear of pain, as well as due to the notion that increased activity can worsen the disease and cause a loss of cartilage, thereby worsening the already existing limited function. The pronounced deficit in body activities significantly further affects mental wellbeing, leading to depression and a poorer quality of life, limiting daily and self-care activities. The Wisconsin study indicated that those with arthritis reported lower physical activity, higher coexisting health conditions, and lower perceived general health status than those without disease [15]. The patients recognised that there was a discrepancy between their desires, real needs, and opportunities, and revised their expectations in terms of performing heavy physical work. Also, at the suggestion of the physician, patients began to change their lifestyle, including minimising activities to protect joints, using exercise that is designed to strengthen muscles, and changing their dietary habits.

The correlations between the WOMAC and EQ-5D scales in our study were satisfactory, although the stiffness component on the WOMAC scale had the lowest correlation with the EQ-5D, which is in accordance with other studies [12].

Our study has several limitations. It was designed as a cross-sectional instead of a follow-up study. Another limitation could be the small sample size which may restrict the generalisation of the observed results. Also, data about possible confounders such as smoking, physical activity, and use of analgesics were not collected, and data about comorbidity were not presented in this paper. We did not measure knee medial-lateral instability which is often associated with knee pain.

## 5. Conclusion

The severity of clinical manifestations and radiographic features may affect functional ability and quality of life in knee OA patients living in rural areas and should all be taken into account when making final clinical decisions to direct proper treatment.

## Figures and Tables

**Table 1:** Characteristics of patients with knee osteoarthritis.

Variable	Total (*n* = 100)
Age (years), mean ± SD	64.6 ± 8.4
Males/females, *n* (%)	25/75
Duration of disease (years)	
≤5	46
6–10	33
>10	21
Affection of both knee joints, *n* (%)	92
Body mass index (kg/m^2^), mean ± SD	30.0 ± 3.0
Kellgren–Lаwrence scale, *n* (%)	
Grade 1, *n* (%)	3
Grade 2, *n* (%)	15
Grade 3, *n* (%)	62
Grade 4, *n* (%)	20

SD: standard deviation.

**Table 2:** WOMAC, VAS, and EQ-5D scores in patients with knee osteoarthritis.

	Mean (SD)
WOMAC pain subscale	10.4 (2.6)
WOMAC stiffness subscale	3.3 (1.3)
WOMAC function subscale	47.8 (7.9)
Visual analogue scale	7.5 (1.1)
EQ-5D subscales	
Mobility	2.03 (0.17)
Self-care	2.00 (0.28)
Usual activities	2.06 (0.28)
Pain/discomfort	2.80 (0.40)
Anxiety/depression	2.02 (0.24)

SD: standard deviation.

**Table 3:** Spearman correlation coefficients between age, duration of diseases, and BMI and WOMAC, VAS, and EQ-5D subscale values.

	Age	Duration of disease	BMI
WOMAC pain scale	0.280^*∗∗*^	0.525^*∗∗*^	−0.109
WOMAC stiffness scale	0.168	0.216^*∗*^	−0.187
WOMAC functional scale	0.336^*∗∗*^	0.397^*∗∗*^	0.029
EQ-5D score			
Mobility	0.162	0.179	0.205^*∗*^
Self-care	0.237^*∗*^	0.273^*∗∗*^	0.058
Usual activities	0.332^*∗∗*^	0.261^*∗∗*^	0.161
Pain/discomfort	0.252^*∗*^	0.504^*∗∗*^	0.065
Anxiety/depression	0.185	0.242^*∗*^	0.148
Visual analogue scale	0.291^*∗∗*^	0.451^*∗∗*^	0.107

^*∗*^Correlation is significant at the 0.05 level; ^*∗∗*^correlation is significant at the 0.01 level.

**Table 4:** Comparison of demographic and clinical characteristic, WOMAC, EQ-5D, and VAS subscores of OA patients between early (Kellgren–Lawrence stage 1-2) and late (Kellgren–Lawrence stage 3-4) radiological stages.

Characteristic	Kellgren–Lawrence stage		
	1 or 2	3 or 4	*p* value
Age	56.0 ± 6.6	69.5 ± 5.3	<**0.001**^*∗*^
Duration of disease	2.3 ± 1.2	4.8 ± 1.2	<**0.001**^*∗∗*^
Body mass index (kg/m^2^)	28.1 ± 2.5	30.4 ± 3.0	**0.004** ^*∗*^
EQ-5D			
Mobility	2.0 ± 0.0	2.0 ± 0.2	0.412^*∗∗*^
Self-care	1.9 ± 0.2	2.0 ± 0.3	0.360^*∗∗*^
Usual activities	2.0 ± 0.0	2.1 ± 0.3	0.303^*∗∗*^
Pain/discomfort	2.5 ± 0.5	2.9 ± 0.3	**<0.001** ^*∗∗*^
Anxiety/depression	2.0 ± 0.3	2.0 ± 0.2	0.711^*∗∗*^
Visual analogue scale	6.7 ± 1.3	7.6 ± 1.1	**0.002** ^*∗∗*^
WOMAC			
Pain	8.9 ± 3.0	10.8 ± 2.4	**0.041** ^*∗∗*^
Stiffness	3.2 ± 1.5	3.4 ± 1.3	0.413^*∗∗*^
Function	42.3 ± 9.7	48.9 ± 6.9	**0.005** ^*∗∗*^

Values are expressed as means ± SD. ^*∗*^According to *t*-test; ^*∗∗*^according to Mann–Whitney *U*-test. Numbers in bold: statistically significant difference.

**Table 5:** Spearman correlation coefficients between EQ-5D and WOMAC subscales.

	Pain	WOMAC stiffness	Function
EQ-5D			
Mobility	0.304^*∗∗*^	0.309^*∗∗*^	0.298^*∗∗*^
Self-care	0.444^*∗∗*^	0.406^*∗∗*^	0.430^*∗∗*^
Usual activities	0.406^*∗∗*^	0.342^*∗∗*^	0.424^*∗∗*^
Pain/discomfort	0.505^*∗∗*^	0.129	0.549^*∗∗*^
Anxiety/depression	0.280^*∗∗*^	0.345^*∗∗*^	0.280^*∗∗*^

^*∗*^Correlation is significant at 0.05 level, ^*∗∗*^correlation is significant at 0.01 level.

## Data Availability

The data that support the findings of this study are available from the corresponding author upon reasonable request.
